# Dr QI - A quality improvement (QI) approach to designing and delivering QI training

**DOI:** 10.1192/bjo.2021.475

**Published:** 2021-06-18

**Authors:** Deepa Bagepalli Krishnan, Victor Ohize, Luke Baumber

**Affiliations:** 1 Institute of Mental Health, University of Nottingham, Nottinghamshire Healthcare NHS Foundation Trust; 2 Nottinghamshire Healthcare NHS Foundation Trust

## Abstract

**Aims:**

To develop and implement a QI training programme for trainees, Trust grade doctors and Consultants in Nottinghamshire Healthcare NHS Trust (NHFT) to enable them to deliver change in practice through acquisition of new knowledge and practical application of skills in QI projects using Model for Improvement.

**Background:**

QI is crucial to improve patient care. Doctors are uniquely placed to input into patient safety and service delivery of healthcare. The skills required to be future clinical leaders and undertake improvement work are not innate and formal teaching and support is required.

What is DrQI?

DrQI is a trainee-led QI teaching programme developed in collaboration with Trainees improving patient safety through QI (TIPSQI) in North West deanery.

**Method:**

A pre-implementation survey amongst doctors in NHFT in February 2019 (33 responses) suggested that 90% of doctors were interested in learning QI and about 48% preferred face-face workshops with support from the QI team.

A list of change ideas were created using a driver diagram with QI education and project support identified as key primary drivers.

PDSA cycles

Nine interactive workshops teaching key QI concepts (based on model for improvement) in NHFT, training more than 100 doctors. A workshop in Derbyshire Healthcare NHS Foundation Trust (70 doctors) and Nottingham University Hospital (20 doctors). Workshops were continually adapted based on qualitative and quantitative feedback. Different formats were tried including virtual sessions, game-based and problem-based learning approaches using small group activities.

**Result:**

Pre-course and post-course questionnaires were used to assess change in understanding of individual components of QI methodology (SMART Aim, Driver diagram, PDSA cycles, outcome and process measures and run charts). Mean pre-course self-assessment score collated from seven QI workshops in NHFT (2019-2020) was 3.3 and mean post-course score was 7.68, showing an improvement in understanding of QI methodology.

Participants were asked to score the relevance (8.4) and quality of teaching (8.4) and the support from the QI team (7.4) on a scale of 1-10 (1 = poor and 10 = excellent). Additional free text feedback was obtained to help us improve the teaching programme.

**Conclusion:**

Collaborative leadership trainee-led initiative to increase the QI capacity. A bottom up approach to complement the top down approach from the Trust QI team. Future steps include further collaboration and expansion of the scheme to other Trusts, Train the trainer sessions and building a network of QI champions.

